# Low-density lipoproteins investigated under high hydrostatic pressure by elastic incoherent neutron scattering

**DOI:** 10.1140/epje/i2017-11558-8

**Published:** 2017-07-26

**Authors:** J. Peters, N. Martinez, B. Lehofer, R. Prassl

**Affiliations:** 1 Univ. Grenoble Alpes, LiPhy, F-38044 Grenoble, France; 2 Institut Laue Langevin, F-38000 Grenoble, France; 3 Univ. Grenoble Alpes, IBS, F-38000 Grenoble, France; 4 Institute of Biophysics, Medical University of Graz, A-8010 Graz, Austria

## Abstract

Human low-density lipoprotein (LDL) is a highly complex nano-particle built up of various lipid classes and a single large protein moiety (apoB-100) owning essential physiological functions in the human body. Besides its vital role as a supplier of cholesterol and fat for peripheral tissues and cells, it is also a known key player in the formation of atherosclerosis. Due to these important roles in physiology and pathology the elucidation of structural and dynamical details is of great interest. In the current study we drew a broader picture of LDL dynamics using elastic incoherent neutron scattering (EINS) as a function of specified temperature and pressure points. We not only investigated a normolipidemic LDL sample, but also a triglyceride-rich and an oxidized one to mimic pathologic conditions as found under hyperlipidemic conditions or in atherosclerotic plaques, respectively. We could show that pressure has a significant effect on atomic motions in modified forms of LDL, whereas the normolipidemic sample seems to cope much better with high-pressure conditions irrespective of temperature. These findings might be explained by the altered lipid composition, which is either caused through elevated triglyceride content or modifications through lipid peroxidation.

## 1 Introduction

Low-density lipoproteins (LDL) are naturally occurring macromolecular assemblies of various lipids and a single protein component, named apolipoprotein B-100 (apoB-100). ApoB-100 stabilizes the structure of the lipid nano-assembly and triggers the function of LDL in human blood circulation. The major role of LDL in humans is the transport of cholesterol and fat to tissues and cells. Apart from its vital role in physiology, LDL is intimately involved in the progression of cardiovascular diseases, in particular atherosclerosis. Chemical modifications of LDL, *e.g.* through oxidation, cause an accumulation and retention of oxidized LDL in the subendothelial space for ingestion by macrophages, which are subsequently transformed into foam cells. In combination with inflammatory reactions LDL retention in the arterial wall constitutes the first stage of atherosclerosis. Atherosclerosis dramatically increases the risk for myocardial infarction and stroke, which are amongst the major causes of morbidity and mortality in Western civilization resulting in substantial economic burden imposed on health care systems [1]. The interaction of surface-located apolipoproteins with specific cellular receptors indeed determines whether triglycerides and cholesterol will be added or removed from the lipoprotein transport particles [2]. According to these functionalities, it is a common use to distinguish between “bad” and “good” cholesterol when designating LDL cholesterol and high-density lipoprotein (HDL) cholesterol, respectively. However, certain blood levels of both HDL and LDL are essential to maintain health. LDL’s structure was investigated exhaustively by small-angle scattering techniques and cryo-electron microscopy [3–7], giving rise to a rough representation of the nano-particle. It has an ellipsoidal shape with radii varying between 87 and 123 Å for the different axes [8]. Contrary to a cell bilayer membrane, its surface is formed by a phospholipid monolayer, and the hydrophobic core is filled with cholesteryl esters and triglycerides. According to the environmental temperature and the actual core composition, the core is more or less ordered in layers [7]. Indeed, LDL nano-particles exhibit phase transitions, whose exact temperature *T_m_* depends on the samples’ core composition [9,10].

The molecular dynamics of biological systems is as important as the structure for the proper functioning of bio-systems [11]. Lipoproteins have shown a distinct dynamical behaviour as a function of temperature [12]. However, temperature is only one important thermodynamical variable, therefore to completely describe a system its dependence on pressure has to be taken into account as well [13]. Such pressure experiments are still scarce, especially in combination with neutron scattering techniques, mainly due to technological challenges. Recently, we investigated LDL particles under high hydrostatic pressure (HHP) conditions by quasi-elastic and elastic incoherent neutron scattering [8] and found significant differences in dynamics and shape of LDL according to the actual core composition. These studies were performed at body temperature (310 K), only. Here, we present elastic incoherent neutron scattering (EINS) results as a function of temperature at the two extreme pressure points investigated before, *i.e.* 20 bar and 3000 bar. Temperature and pressure are expected to have opposing effects. In fact, temperature increases the thermal energy and the volume of a system and thus its mobility, whereas pressure decreases the available volume and thus the flexibility according to Le Châtelier’s principle [14]. We compared the dynamics of LDL samples having a normolipidemic core composition (N-LDL) to LDL particles with a triglyceride-rich core composition (TG-LDL) mimicking LDL particles usually found in patients with hyperlipidemia and a proatherogenic oxidatively modified LDL sample (Ox-LDL).

## 2 Experimental details

### 2.1 Sample preparation and characterization

LDL was isolated from human blood plasma by applying a multiple-step density gradient ultracentrifugation [12]. Blood plasma was obtained from the Department of Blood Group Serology and Transfusion Medicine of the University Hospital Graz (Graz, Austria) after written informed consent, according to a protocol approved by the Institutional Review Board of the Medical University of Graz. The blood plasma was free of pathogens (HBV, HCV, HIV). The isolated LDL was extensively dialyzed against buffer solution (10 mM NaPi buffer (1.44 g/L Na_2_HPO_4_ · 2 H_2_O, 0.26 g/L KH_2_PO_4_), 0.1% EDTA, *p*H 7.4), concentrated with Amicon Ultra-15 centrifugal filter units (cut-off 100 kDa) and characterized for its protein and lipid composition. Protein concentration was determined with BCA protein assay kit (Thermo Fisher Scientific, Waltham, MA, USA). The further biochemical composition was determined with colorimetric enzymatic assay kits (DiaSys Diagnostic Systems, Holzheim, Germany) as described previously [15]. Protein integrity was checked with SDS-PAGE.

For the oxidation process LDL was rebuffered in an oxidation buffer (10 mM NaPi, 0.9% NaCl, *p*H 7.4) which did not contain EDTA to enable the oxidation with CuCl_2_. 34 nmol CuCl_2_/mg apoB-100 protein were used to oxidize the sample at 310 K. The oxidation was monitored simultaneously at a spectrophotometer through the increase of absorption from conjugated dienes at 234 nm as described by [16]. The process was stopped after 135 min in the middle of the propagation phase through the addition of EDTA. Finally, the samples (N-LDL, TG-LDL and Ox-LDL) were rebuffered to the measurement buffer (10 mM NaPi, 0.1% EDTA, *p*H* 7.4 (*p*D 7.8) in D_2_O). The *p*H was measured with an electrode in the D_2_O solution and adjusted to *p*H* 7.4. The *p*H* value is a *p*H related quantity representing a direct reading in a D_2_O solution of a H_2_O-calibrated *p*H-meter. The conversion of *p*H* into *p*D is then accomplished by adding a constant of 0.4 *p*H units [17]. The chemical compositions of the investigated LDL samples are provided in table 1.

**Fig. 1:**
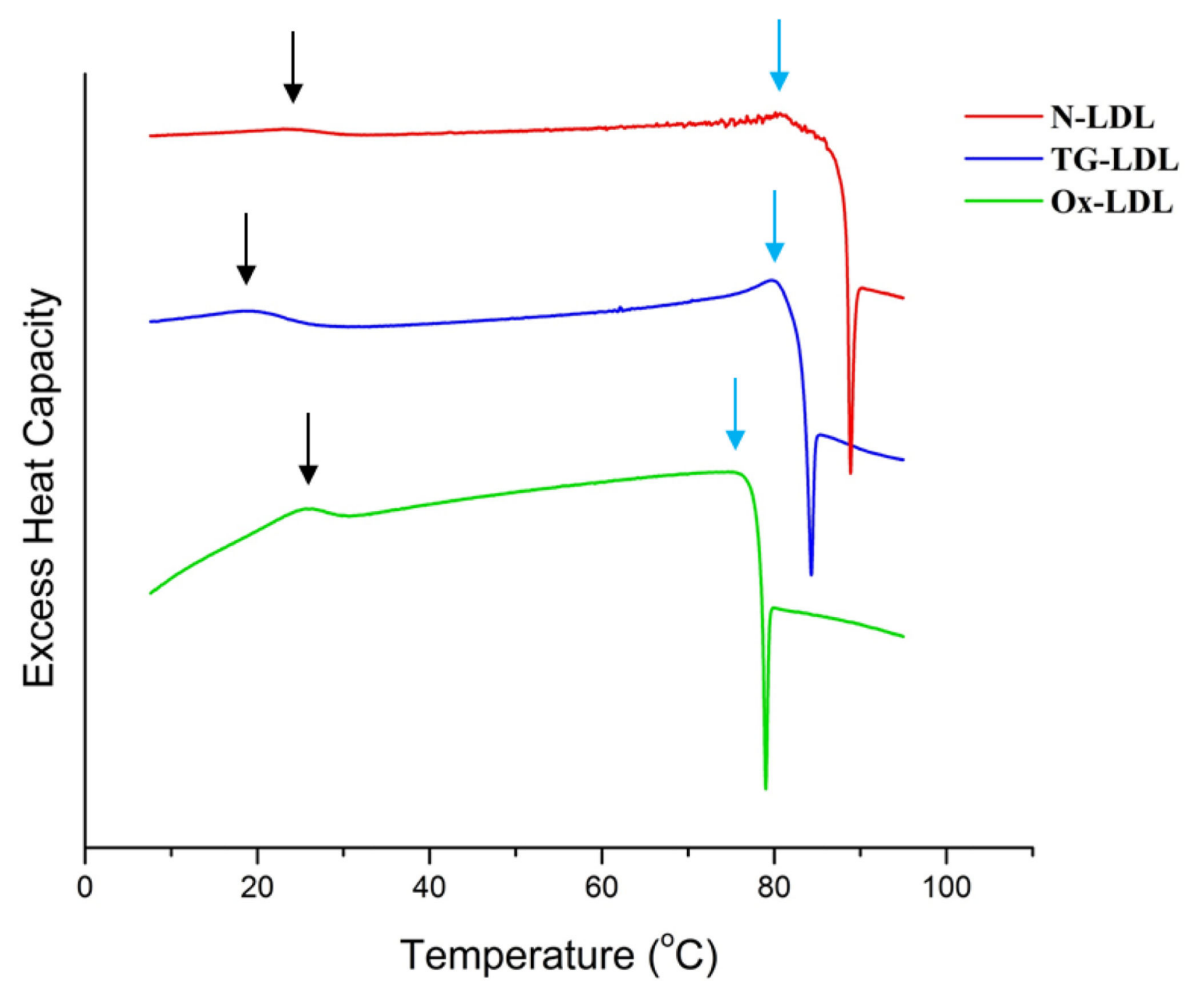
Buffer-subtracted DSC curves (shifted vertically for better visibility) with lipid phase transition temperatures *T_m_* (indicated through black arrows) and the endothermic protein denaturation peak (indicated through blue arrows).

To determine the transition temperatures *T_m_* corresponding to the reversible lipid phase transition, and the protein denaturation temperature of apoB-100, differential scanning calorimetry (DSC) scans were performed with a scan rate of 60 °C/h. Figure 1 shows the buffer-subtracted scans converted into heat capacity *C_p_* as a function of temperature. We found transition temperatures of *T_m_* = 23.5 °C for N-LDL, *T_m_* = 19.1 °C for TG-LDL and *T_m_* = 25.6 °C for Ox-LDL. Note, that *T_m_* varies with the lipid composition of the LDL particles. Usually *T_m_* is found around 25 °C or higher [10] as was the case for N-LDL and Ox-LDL, while the lower *T_m_* of TG-LDL is typical for LDL with a high TG content. The endothermic protein denaturation peaks were around 80 °C for N-LDL and TG-LDL. For Ox-LDL the protein denaturation peak was diminished and shifted to lower temperatures, reflecting protein damage during the oxidation process [18]. The exothermic transition seen as drop in the heat capacity curve at higher temperature is due to particle aggregation and sedimentation. Again, the temperature is lower for oxidized LDL.

### 2.2 Neutron scattering experiments

The EINS experiments, which permit to follow averaged atomic motions, were operated on the thermal backscattering spectrometer IN13 [19] at the Institut Laue Langevin (ILL) in Grenoble/France. The instrument has an energy resolution of 8 *μ*eV, corresponding to a time window of about 100 ps. It therefore allows to see internal motions of macromolecules. Elastic scattering data, corresponding to the situation where no energy is exchanged but the neutrons are deviated from their initial direction giving rise to a variation of momentum (designated as *Q*) were analyzed through the scattering function *S*(*Q*, 0 ± *ΔE*), where *ΔE* designates the instrumental resolution. Assuming a normal distribution of the atoms around their equilibrium position it reduces in terms of the Gaussian approximation [20] to

(1) $$S(Q,0\pm \Delta E)\approx \text{exp}\left(-\frac{1}{3}Q^{2}\langle u^{2}\rangle \right).$$

Here 〈*u*^2^〉 are the average atomic mean-square displacements (MSD). The MSD values are obtained for each temperature/pressure point from the slope of the logarithm of the scattered intensities *S*(*Q*, 0 ± *ΔE*) plotted *versus Q*^2^ according to

(2) $$\langle u^{2}\rangle =-3\frac{\partial \text{ln}S(Q,0\pm \Delta E)}{\partial Q^{2}}.$$

This approximation is strictly speaking only valid for *Q* → 0, but can be extended to 〈*u*^2^〉*Q*^2^ < 1 [21]. Accordingly, in this study the fit range was restricted to the low *Q*-range between 0.5 Å^−1^ and 2.0 Å^−1^. The incoherent neutron scattering intensity is dominated by the signal arising from hydrogen. This is due to the hydrogen incoherent scattering cross section, which is one order of magnitude larger than that of all other atoms usually occurring in biological matter, and also of its isotope deuterium [22]. The technique thus probes average dynamics, because hydrogen atoms are almost uniformly distributed in the sample and representative for the molecular sub-groups to which they are bound. Thus when using D_2_O for the surrounding water, as done in our case, its signal is negligible compared to the signal arising from the sample itself.

A high-pressure equipment dedicated to neutron scattering experiments of biological samples in solution has been developed over the last years at the ILL [23]. It consists of a high-pressure stick, which can be placed in the cryostats or cryofurnaces of the instruments, a pressure controller, which allows a remote control and permanent adjustment of pressure, and a cylindrical pressure container built of a high-tensile aluminum alloy withstanding pressures up to 6 kbar (600 MPa). The HHP sample holder has an outer diameter of 15 mm and an inner diameter of 6 mm, thus the thick Al walls absorb much more neutrons than standard flat sample holders. Accordingly, each pressure and temperature point was measured for at least 10 hours to ensure a sufficient signal-to-noise ratio. We probed the samples at pressure points of 20 bar and 3 kbar and at temperatures of 280 and 310 K. Moreover, we recorded a temperature point close to the lipid phase transition, that is to say at 297 K (24 °C) for N-LDL, at 292 K (19 °C) for TG-LDL and 300 K (27 °C) for Ox-LDL. For correction and normalization purposes, the empty cell, the buffer (10 mM NaPi, 0.1% EDTA in D_2_O) and a vanadium rod (a completely incoherent scatterer) were probed under similar conditions. Absorption corrections based on the formula of Paalman-Pings [24] were applied and the data reduction was carried out using the LAMP software available at the ILL [25]. All samples were normalised in the same way to give comparable absolute intensities.

## 3 Results and discussion

**Fig. 2:**
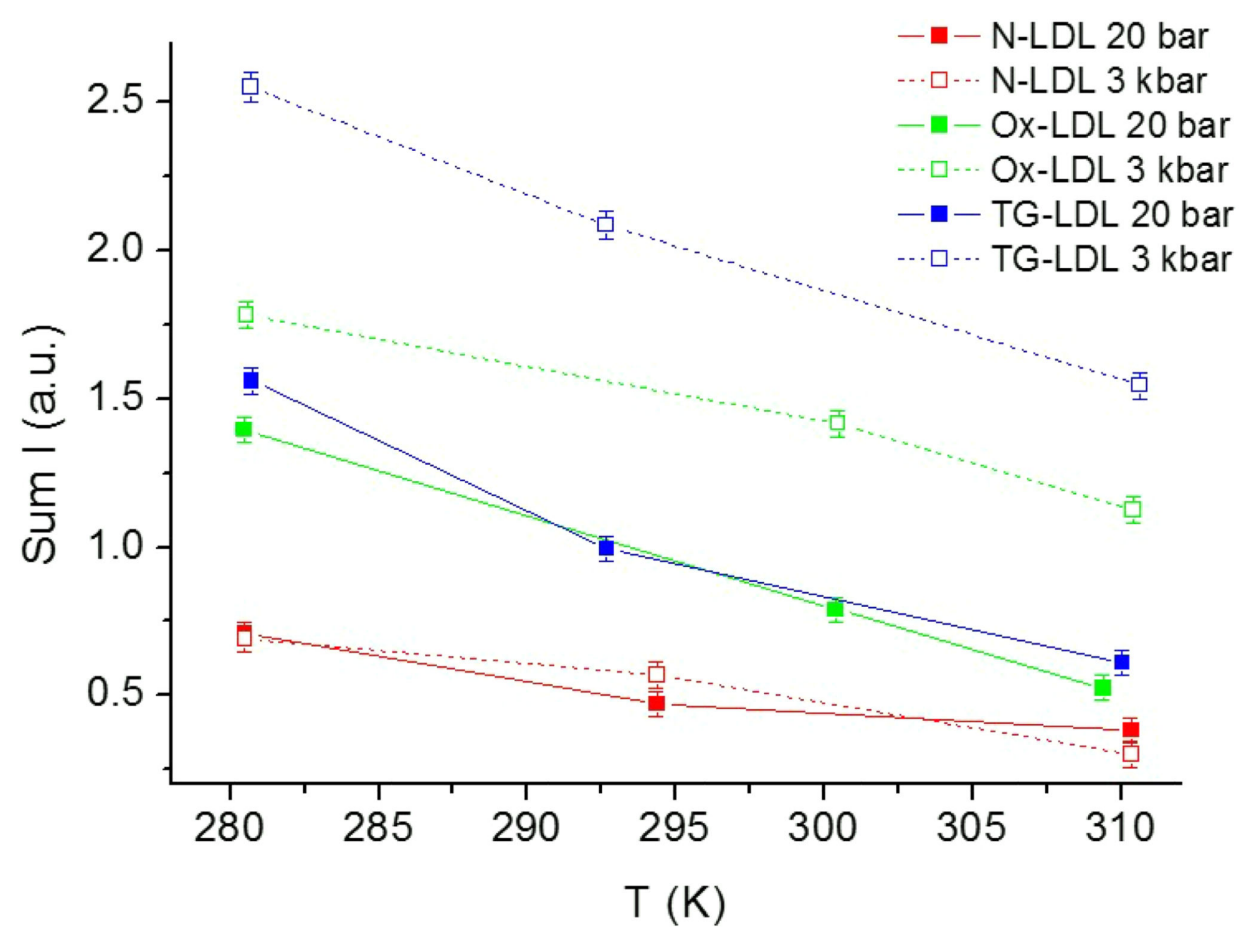
Neutron intensities summed over all scattering angles as a function of temperature for both pressure points. Lines are guides to the eyes.

We first summed the neutron intensities over all scattering angles corresponding to *Q*-values from 0.2 to 4.5 Å^−1^. This operation is not restricted by any approximation and gives lower error bars (see fig. 2). We showed that the summed intensities are inversely proportional to the square root of the MSD [26]. Here we distinguish two effects: i) the summed intensities decrease almost linearly with temperature and ii) they increase with pressure except for N-LDL, for which the intensities remain constant within error bars. The intensity increases when the atomic motions are slowed down and thus more neutrons are scattered within the instrumental time window. On the contrary a lower intensity is an indication for a higher flexibility of the sample.

**Fig. 3:**
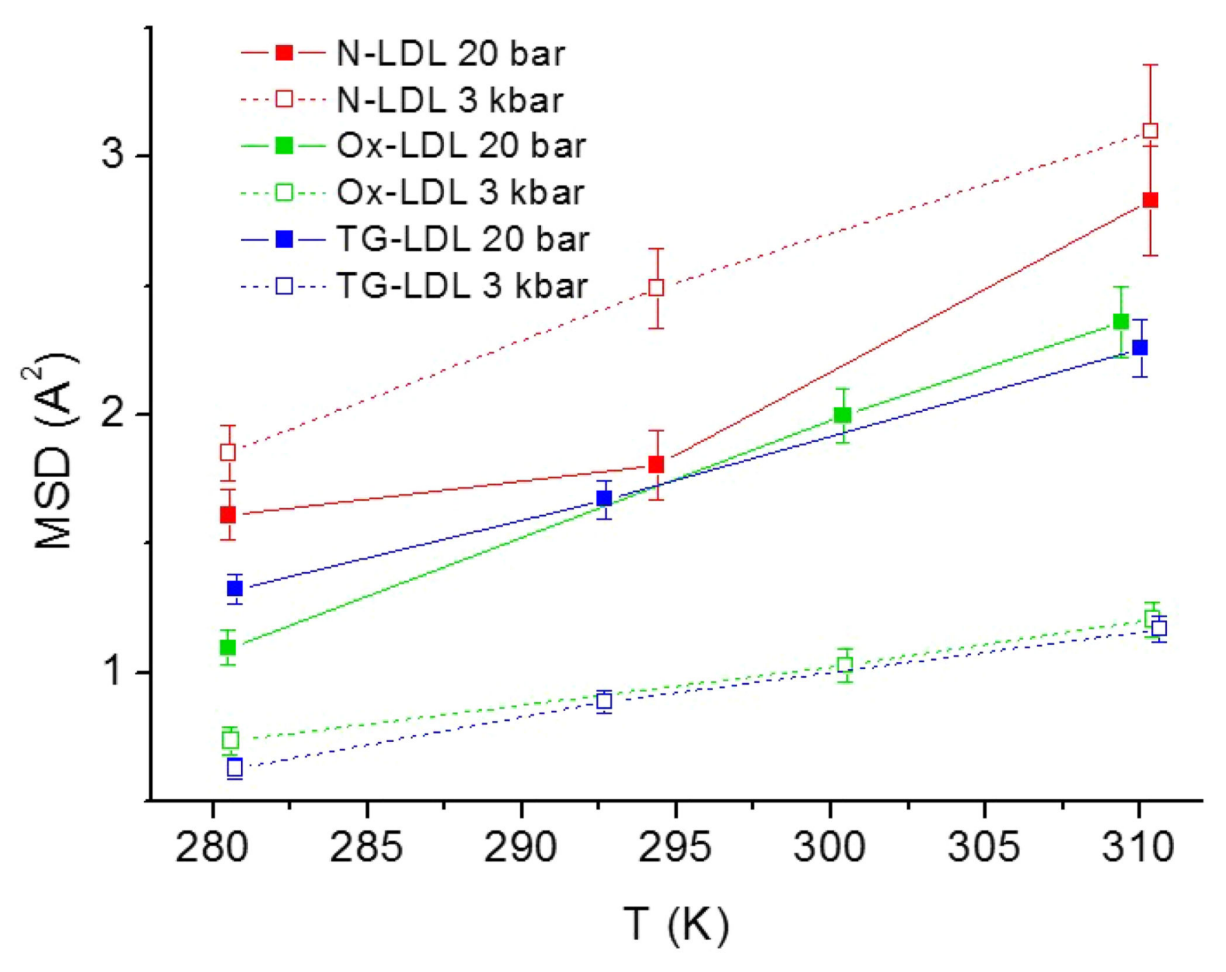
Atomic MSD extracted in the *Q*-range between 0.5 Å^−1^ and 2.0 Å^−1^ as a function of temperature for both pressure points. Lines are guides to the eyes.

Similar results can be extracted from the MSD (see fig. 3): i) the MSD values are all increasing with temperature and ii) they are decreasing at higher pressure, again except for N-LDL for which the MSD values are even slightly increasing with pressure. Therefore, temperature and pressure have clearly different effects: temperature raise increases the thermal energy within the sample, which seems to be roughly homogeneously distributed over all atoms and increases their motions almost equally. In contrast, high pressure specifically affects the different samples, sometimes increasing and sometimes decreasing the flexibility. It seems that the dynamic behavior under pressure of the two LDL particles mimicking pathological conditions is similar and is in clear contrast to the normolipidemic form. This means that temperature has a comparable effect on all samples, whereas pressure seems to specifically induce some structural rearrangements in the modified samples. These effects might be caused by an altered compressibility of surface and core lipids as well as a conformational change of the protein moiety apoB-100 induced by either peroxidation processes or a different core fluidity due to an elevated TG level.

Overall, the MSD of N-LDL are very high compared to other biological macro-molecules [27] and to the MSD of LDL hydrated powder as described in [12] (N.B.: We used a different convention in the two cited publications taking into account a factor 6 in eqs. (1) and (2) instead of 3 as done here, which enhances the differences even more.). Indeed, lipids are known for their high flexibility (about 2.8 Å^2^ at 305 K for DMPC membranes when accounting for the factor 3 instead of 6, see [28]) and in the current experiment the sample was in solution, which increases the flexibility even more as whole molecule’s diffusion could be added [29]. Below and above the phase transition temperature, the MSD of N-LDL at low and high pressure are almost within error bars, but they differ significantly at the phase transition temperature. This is not surprising as fluctuations of any size occur close to a phase transition [30]. However, it is rather surprising that the normolipidemic particle resists so well to HHP application and becomes even slightly more mobile at higher pressure as all MSD points lie above the corresponding value at ambient pressure in contrast to the two other samples. A similar behavior was already detected in our earlier work [8], that is to say that N-LDL coped very well with pressure with respect to its molecular dynamics and its structure.

The modified forms, Ox-LDL and TG-LDL, seem to be much more sensitive to pressure application, which tends to reduce the atomic motions in accordance with Le Châtelier’s principle by a factor of at least two. Triglycerides are indeed much softer than cholesteryl esters and their quantity is enhanced in the lipid core of TG-LDL (see table 1). This hydrophobic core undergoes a phase transition from an ordered smectic-like phase to a more disordered isotropic phase at a certain *T_m_* defined by its exact core composition. This phase transition temperature is highly sensitive to very small changes in the core composition, more precisely to changes of the TG content in the core. Higher TG contents lead to lower transition temperatures. This could also be shown in our study (see fig. 1), where the higher TG content leads to a lower *T_m_*. Therefore it is quite evident that such small changes in the core composition also have significant effects on the dynamical behavior, especially under high-pressure conditions. This might be the reason why the atomic motions in TG-LDL are reduced by pressure more intensively, when a higher amount of triglycerides is present in the lipid core.

The modifications occurring during oxidation might have a similar effect. In the propagation phase of LDL oxidation lipid radicals are generated and start a chain reaction of lipid peroxidation, which leads to the formation of lipid peroxyl and lipid alkoxyl radicals resulting in the formation of conjugated dienes and lipid hydroperoxides [31, 32]. While the core lipid organization is less affected by oxidation, the protein moiety becomes destabilized [18]. Conformational changes of apoB-100 already occur at early stages of oxidation. It could be shown that changes occur predominantly in the *β*-sheet regions of the protein and even before LDL peroxidation is initiated [31]. Such changes certainly influence flexibility and compressibility and consequently the dynamical behavior. Likewise, the oxidatively modified acyl chains of the phospholipids in the surface monolayer are less densely packed and ordered. Hence, it is reasonable to assume that the outer layer of LDL becomes softer leading to a higher compressibility at high pressure. However, neutron scattering averages over all H-atoms within the sample, so the various contributions cannot be distinguished further in more detail.

This is the first study that gives insight into the dynamical behavior of normolipidemic LDL compared to the modified forms using EINS. At the present state, we are not able to draw medical conclusions from our findings. We want to remind that the two modified forms of LDL only mimic pathologies, but are not derived from real patients. Therefore, we can only suggest some very tentative statements: It appears that the normolipidemic form of LDL resists very well to HHP application and its mobility is by trend even higher under pressure than without. Both modified forms of LDL have reduced MSD under HHP application and their thermal behavior is very similar to each other. However, the slope of the MSD as a function of temperature is reduced under high pressure. This means that pressure damps the effect that thermal energy has on the MSD.

## 4 Conclusions

Here we report on dynamic data of lipoproteins as a function of temperature and pressure using elastic incoherent neutron scattering. We chose LDL with a normolipidemic composition and two modified forms of LDL to mimic pathological conditions. Triglyceride-rich LDL characteristic for hyperlipidemia and oxidatively modified pro-atherogenic LDL were investigated. As LDL particles show a lipid phase transition, we measured the samples below, at and above the phase transition temperature at two extreme pressure points of 20 and 3000 bar. In accordance with our recent findings [8], normolipidemic LDL seems therefore to be the optimized form of LDL, coping well with harsh external conditions and staying flexible. On the contrary, for both modified forms of LDL the MSD is strongly reduced under pressure and the expected increase of MSD with increasing temperature is also damped under pressure. These modified particles are therefore much less adapted to external extreme conditions and partly lose their flexibility. We assume that this behaviour is due to the altered lipid profile which might be correlated with LDL functionality.

## Figures and Tables

**Table 1:** Chemical compositions (% w/w of total LDL mass) of normolipidemic N-LDL, triglyceride-rich TG-LDL and oxidized Ox-LDL. Values are means ± SD of duplicate or triplicate determinations of each component.

Component	N-LDL	TG-LDL	Ox-LDL
Protein	21.0 ± 3.1	16.6 ± 0.2	16.1 ± 1.0
Phospholipids	20.6 ± 2.3	21.9 ± 1.7	22.5 ± 3.6
Unesterified cholesterol	9.2 ± 0.7	7.0 ± 0.8	9.1 ± 0.7
Cholesteryl esters	41.4 ± 1.0	44.4 ± 0.7	44.8 ± 1.3
Triglycerides	7.8 ± 0.5	10.1 ± 0.8	7.5 ± 0.1
